# Predictive Behavior of a Computational Foot/Ankle Model through Artificial Neural Networks

**DOI:** 10.1155/2017/3602928

**Published:** 2017-01-30

**Authors:** Ruchi D. Chande, Rosalyn Hobson Hargraves, Norma Ortiz-Robinson, Jennifer S. Wayne

**Affiliations:** ^1^Department of Biomedical Engineering, Virginia Commonwealth University, 401 West Main Street, P.O. Box 843067, Richmond, VA 23284-3067, USA; ^2^Department of Electrical Engineering, Virginia Commonwealth University, 601 West Main Street, P.O. Box 843072, Richmond, VA 23284-3072, USA; ^3^Department of Mathematics & Applied Mathematics, Virginia Commonwealth University, 1015 Floyd Avenue, P.O. Box 842014, Richmond, VA 23284-2014, USA

## Abstract

Computational models are useful tools to study the biomechanics of human joints. Their predictive performance is heavily dependent on bony anatomy and soft tissue properties. Imaging data provides anatomical requirements while approximate tissue properties are implemented from literature data, when available. We sought to improve the predictive capability of a computational foot/ankle model by optimizing its ligament stiffness inputs using feedforward and radial basis function neural networks. While the former demonstrated better performance than the latter per mean square error, both networks provided reasonable stiffness predictions for implementation into the computational model.

## 1. Introduction

Computational models of diarthrodial joint function depend on accurate reproduction of bony and soft tissue characteristics. Certain characteristics may be readily acquired from imaging modalities while others require experimentation. This is particularly challenging when developing patient-specific models for which soft tissue material properties, for example, cannot be easily obtained. In such cases, existing literature is referenced and best estimates serve as input into the computer model. Further complicating model inputs are soft tissue properties that have not or cannot be determined experimentally and for which the properties of known tissues with similar functions are applied. In either situation, data from the literature usually includes a wide range of values or large standard deviations, impacting the efficacy of the model. Because such tissue inputs directly affect the function of the computational model, the model's predictive capability is only as successful as the information provided to the model. Thus by improving the accuracy of the inputs, the performance of the model will be improved. In the current work, a means of optimizing a model's inputs, specifically ligament stiffness, was sought for the greater purpose of enhancing the predictive ability of the computational model.

To optimize the ligament stiffness, artificial neural networks (ANNs) were considered. ANNs are mathematical models in which interconnected computational units or neurons [1, 2] are utilized to “learn” relationships among data [2, 3]. To learn this relationship, ANNs attempt to minimize a given cost function by using an iterative process, that is, learning rule [3, 4], to continually adjust system weights until a target is achieved [1]. Once it learns the relationship between known input-output data, the ANN can then apply this knowledge to similar, never-before-seen data to predict an output [1, 3]. Therefore, ANNs are capable of generalizing, meaning they are able to determine a reasonable output based on learned knowledge [1, 5]. Also, they can be utilized without knowing much about the input-output relationship a priori [1], an advantage over statistical regression in which a mathematical formulation for the problem is known or assumed to be known [1, 5]. Additionally, ANNs are applicable to nonlinear problems [1]. Generally, neural networks are useful in several applications including pattern and image recognition, classification, and curve-fitting, and various examples of these applications can be found within the biomedical field, including musculoskeletal modelling [1, 3–12].

**Figure 1:**
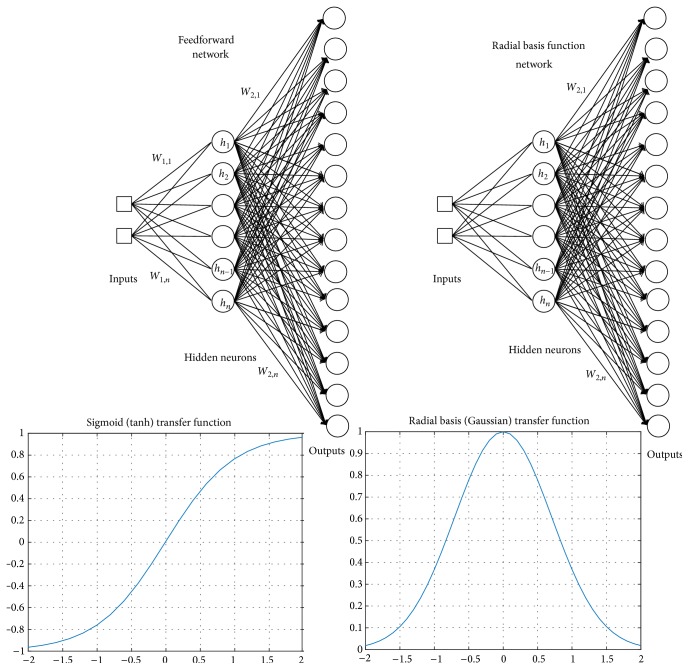
General structure for feedforward (FFN) and radial basis function (RBFN) networks. Sigmoidal and Gaussian transfer functions appear in the hidden layers' neurons for the FFN and RBFN, respectively. (*x* = input; *w*_*in*_ = *n*th weight in layer *i*; *y* = output; *h*_*m*_ = *m*th hidden neuron) (adapted from [1, 13]).

Different types of ANNs exist and determination of which type to use is usually dependent on a given project's application [5]. Because the current work falls under function approximation, the following descriptions will focus on feedforward (FFN) and radial basis function (RBFN) networks (Figure 1). Structurally, the basic unit of any ANN is the neuron or node. In the case of a FFN, each input is first multiplied by a weight factor, and then all weighted inputs are summed together along with a bias prior to passing through the activation function. The activation function, also referred to as a transfer function, can take various forms (e.g., linear, piece-wise, or sigmoidal [1, 6, 13]), but in each case its purpose is to limit the output of a given neuron within a certain range [1]. As for RBF networks, source inputs are not weighted; rather, these inputs are passed through a distance function essentially calculating how far the vector formed by the input data is from a predetermined vector of the same dimensionality, known as a “center.” The result of this calculation is passed through a radial basis function, such as a Gaussian function, and the produced value is the output of the neuron [1, 14].

To form a network, several of these neurons may be arranged together within layers thus defining the network's architecture, where the nodes of each layer feed their output to those of the next layer. A neural network has (a) an input layer of nodes that are fed with the inputs from the environment, (b) an output layer, whose nodes output the processing result of a given input to the environment, and (c) one or more layers arranged between the input and the output layers, which do not interact directly with the environment. One such architecture is known as a multilayer feedforward network, which includes at least one hidden layer with hidden neurons inserted between inputs and an output layer [1]. An RBFN is an example of a multilayer feedforward network as it contains precisely one hidden layer [5]. A hidden layer, with hidden neurons named as such due to their location, is any layer that does not directly contribute to the final output of the network, and so it can be viewed as an intermediate layer. The purpose of these hidden layers is to add an additional means of feature extraction so as to aid the network in deciphering more complex input-output relationships, perhaps one of nonlinear nature [1, 5]. The final relationship between known input-output data is dictated by the specific architecture (number of hidden layers and their respective sizes and type of nodes) of the network, as well as the node parameters determined from learning (e.g., node weights and biases for FFNs and centers and widths of RBFs for RBFNs) [5].

Given their advantages and applications within the biomedical arena, and the means by which they function, ANNs were considered an appropriate and noninvasive means to further study the foot/ankle complex. Specifically, two types of artificial neural networks, feedforward and radial basis function networks, were used to predict ligament stiffness for a computational foot/ankle model in order to improve the computer model's predictive capability.

## 2. Methods

Computational models of Adult Acquired Flatfoot Deformity (AAFD), created in SolidWorks 2007 (SolidWorks Corp., Concord, MA), were generated during previous work [15], and subsequently, one of these models was used in the current research. AAFD is a degenerative foot condition that results in joint misalignment and subsequent pain and discomfort for the patient. The condition's development follows posterior tibial tendon dysfunction with several ligaments implicated in the disease including the spring and deltoid ligaments, plantar tissues, and the talocalcaneal interosseous ligaments [16].

The models developed by Spratley et al. [15] were driven by patient-specific anatomy obtained from magnetic resonance imaging (MRI), muscle contractions represented as a percent of body weight, and ligament stiffness included as spring elements. For those ligaments implicated in AAFD, graded stiffness values were assigned per clinician evaluation. Following load application, represented as single-leg stance, the model kinematics (relative bony angles and distances [15, 17, 18]) were appropriately representative of function, but further improvement was sought.

Because the computer model's inputs dictate its function, model performance would be improved with support for input magnitude. This study focused on enhancing ligament stiffness assignment via application of an ANN to establish the relationship between stiffness and foot/ankle kinematics. Just as other musculoskeletal studies utilized data from computational models for their input-output training data [8, 9], here, a single patient-specific model was used to generate input-output pairings, and then network architecture was determined. First, the model's original ligament stiffness values were varied up or down by a given percentage under the originally prescribed loading (i.e., single-leg stance loading) and the resulting kinematics recorded. Because ANNs require large amounts of data [5], additional input-output pairs were created from the computer model itself such that the networks could learn from a wide range of training scenarios how ligament stiffness could influence kinematic parameters. Furthermore, since the inputs to each foot/ankle model developed in the previous work [15] were patient-specific, the generated training data could originate only from that single patient's model which was to be optimized. Therefore, variations were carried out in 5% increments and these adjustments were applied to one or more of three ligament groupings. The three groupings consisted of those ligaments implicated in AAFD and each group was separated from one another generally by ligament location (i.e., all medial ligaments comprised one grouping). Also, additional input-output pairs were created by varying stiffness from what would be considered normal values for the given ligaments. In total, approximately 160 data pairs were created to use for ANN training. It is important to note here that while the computer model's inputs were ligament stiffness and its outputs comprised kinematic measures, the input-output definition for the ANNs was reversed. Therefore, the ANNs were trained on known kinematic-stiffness pairings with the goal of predicting ligament stiffness values for assessment of network performance.

**Figure 2:**
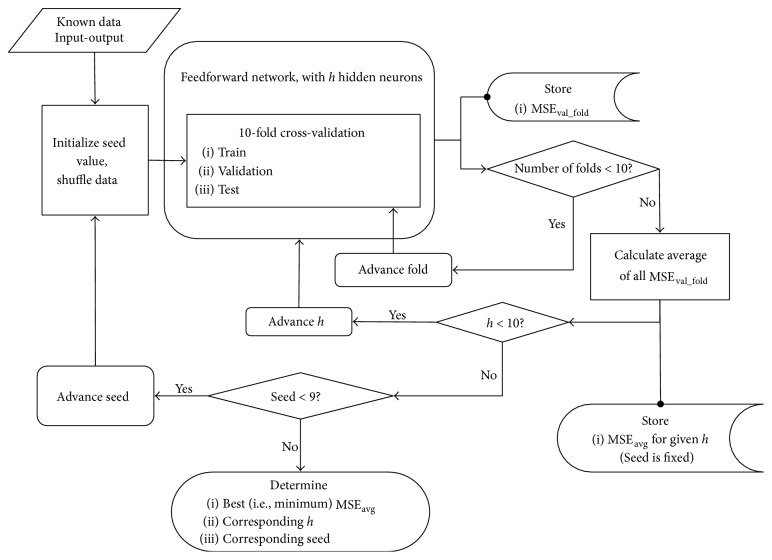
Training process, feedforward network. The flowchart depicts the nested * for* loop structure of feedforward network training, which ultimately chooses the optimal network of those tested based upon minimum mean square error (MSE). Along with this minimum error, the number of hidden neurons and seed value corresponding to the minimum error is output.

Because the task of predicting ligament stiffness is considered a curve-fitting problem, both feedforward and radial basis function networks were explored in the current work. Using MATLAB R2015a (The MathWorks, Inc., Natick, MA), multiple FFNs with a single hidden layer, two inputs (i.e., kinematic angles), and fourteen outputs (i.e., ligament stiffness elements) were created, each with varying hidden layer sizes and weight initializations. Hidden and output layer neurons included tan-sigmoid (steepness parameter of 1) and linear transfer functions, respectively. Beginning with a single hidden neuron, the size of the hidden layer was incrementally increased by one to a maximum of ten hidden neurons in order to determine the ideal network size (Figure 2). Determination of the optimal number of hidden neurons and the seed value of the random number generator (rng), the latter of which guaranteed reproducibility of results and effectively established optimal weights for the FFN, was accomplished using a series of nested “for” loops. Furthermore, 10-fold cross-validation was implemented to facilitate network selection. Generally, cross-validation is a methodology in which the known dataset is first divided into a given number of folds (in this case 10). A single fold is left out as test data, while the remaining folds of data are pooled together to train the network. Following training, the network is tested with the previously left out fold and the ANN's performance is assessed. Then, the data fold is placed back into the training subset while a second fold is left out as test data. This process is repeated until each of the folds is left out once as test data; finally, the overall performance of the network is determined by averaging all folds' test performances.

For every layer size of the FFNs created here, cross-validation was adapted to generate training, validation, and test subsets, while performance was evaluated using mean square error (MSE). MSE was calculated as shown in (1) in which *N* is the number of input-output pairs, *t* is the target value (stiffness), and *a* is the network prediction [13]. Per (1), MSE is minimized as network predictions move toward target values; therefore, MSE values closer to 0 represent better-performing networks. (Note: because MSE sums the squared differences between targets and predictions, error values may favor larger elements over smaller elements in dataset that include varying scales for inputs and/or outputs. As a result, input-output data may be standardized to minimize such effect. In this study, inputs and outputs were standardized within a range of [−1,1] prior to the calculation of MSE.)

(1) $$\begin{array}{c} MSE=\frac{1}{N}\overset{N}{\underset{i=1}{\sum }}{\left(\;t_{i}-a_{i}\;\right)}^{2}. \end{array}$$

Average MSE on the validation subset was determined for each combination of hidden neuron number and seed value, and the network corresponding to the smallest of these average performances was selected as the optimal network. Following its selection, MSE of the test data was calculated.

**Figure 3:**
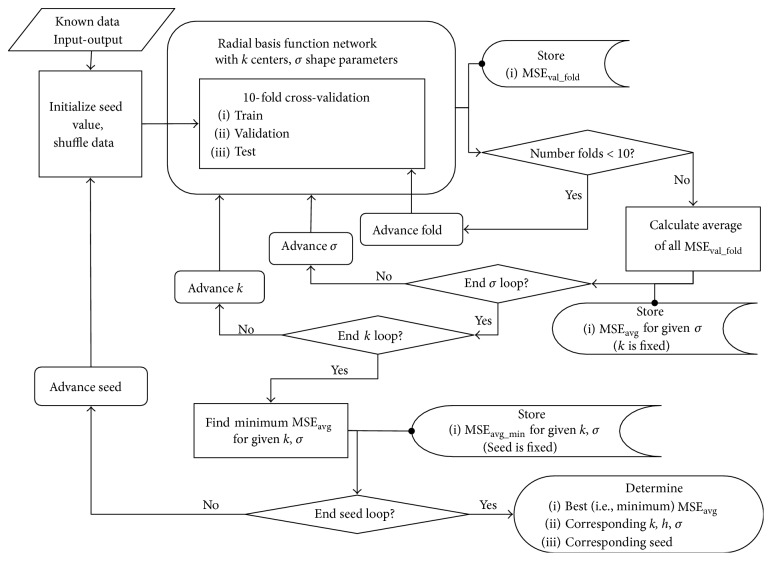
Training process, radial basis function network. Similar to the feedforward network, the radial basis function network also includes a nested * for* loop structure. Here, an optimal network is chosen based on minimum mean square error of each *k*, *σ*, and seed value combination.

To implement the RBFN (Figure 3), training data, center values, and shape parameters were input into a code utilizing a proprietary RBFN function file, developed by Kecman, Ph.D. [19], so as to determine the optimal network. Generally, a cross-validation procedure similar to that just described facilitated data division and network selection. In addition to seed value (which, again, guaranteed reproducibility, and randomly shuffled the known input-output pairs), the optimal number of centers (effectively determining the number of neurons) and shape parameter were selected using embedded “for” loops. Just as with the FFNs, MSE was calculated for each subset of data, and the network corresponding to the smallest average MSE on the validation set was selected as the optimal RBFN. Subsequently, the corresponding center and shape parameters were identified and error on the test set was calculated.

Once defined, the optimal FFN and RBFN structures were each used to predict stiffness values for the computational model (ground truth). For each of the fourteen outputs, the network-predicted stiffness was compared to the originally assigned stiffness values by computing a percent difference relative to the latter of the two values.

## 3. Results

Following training with known kinematic (talo-1st metatarsal and talonavicular angles) stiffness (fourteen components) pairings, optimal FFN and RBFN were chosen, their parameters noted (Table 1), and their performances compared (Table 2). Again, the optimal network was defined as that network which resulted in the smallest MSE among all the networks trained. The optimal FFN was found to have a smaller number of neurons in its hidden layer as compared to the RBFN. For the latter, *k* represented the number of centers in the network. In other words, every *k*th value in the dataset served as a center and thus indicated how many neurons the resulting network had.

Regarding performance error, the FFN had a lower performance (i.e., smaller mean square error) than the RBFN when MSE on the validation sets were compared, while the reverse was true for the test sets. Correlation values, *R*, were similar between the two ANNs.

Following performance comparisons, the optimized networks were used to predict stiffness values for all fourteen ligament components (Table 3). A percent difference was calculated relative to target ligament stiffness (i.e., ground truth) with the FFN and RBFN resulting in maximum absolute differences of about 6% and 29%, respectively.

## 4. Discussion

Predicting the relationship between kinematic behavior of a computational foot/ankle model and soft tissue (ligament) characteristics was accomplished with an artificial neural network. ANNs were chosen for the current work for several reasons. First, the contribution of each ligament alone or in combination with others to a particular kinematic measure was not readily apparent and thus deemed ANNs a desirable approach for honing in on specific stiffness values. Further, theoretically, it has been shown that a multilayer ANN with a single hidden layer can be employed to model any function [2, 5], and so this knowledge also attributed to the selection of ANNs for the current study. Finally, ANNs were pursued as a viable optimization means as various others have applied them to the biomedical field [3–12].

In the musculoskeletal area, ANNs have been used to determine cartilage stress within a computationally modelled knee [8], as well as contact between a computationally modelled femoral component and tibial plateau of a knee implant [9]. In the former example, reaction forces due to cartilage contact simulated in a multibody model served as the inputs and von Mises stresses from a finite element (FE) model served as the outputs during network training. Ultimately, the investigators were able to successfully predict cartilage stress from the neural networks developed [8] as the predictions were similar to their ground truth model. In the latter example, Eskinazi and Fregly [9] utilized an ANN approach to develop a computationally faster and more accurate model to predict contact. Using translations and rotations as inputs and contact forces and torques as outputs observed between the modelled components, a series of ANNs were successfully trained. Subsequently, the ANNs proved to have more accurate contact predictions and managed to output the predictions faster than the investigators' existing surrogate contact model [9]. A third example describes classification of fracture healing in which Kaufman et al. represented both intact and fractured bone with a vibrating, cylindrical beam. The equations describing the behavior of this beam were translated into an electrical model in which admittance values stood for the beam's vibrational characteristics. Subsequently, an ANN was trained on these admittance values (inputs) and classifications representing one of four levels of a healed fracture (outputs). Good ANN performance was observed and the researchers planned to expand the ANN application to human and animal models [10]. Because the preceding examples used ANNs successfully to complement biomechanical studies, their use for stiffness optimization was considered applicable to the current work and implemented in a preliminary study [20].

In the case of the networks trained here, the validation set performance of the FFN was better in comparison to that of the RBFN. Strictly from a comparison standpoint, if a single network was to be chosen between the two types, the FFN would be favored due to its slightly lower MSE value on the validation set despite the fact that its test set performance was higher than that of the RBFN. Generally, however, as the two networks' mean square errors were similar, it may be that either of the networks would provide reasonable stiffness predictions for the particular model used in this study.

To further investigate our optimal networks' performances, correlation was observed on the entire dataset (*N* = 163) (Table 2); good correlations were found between targets and predictions for both networks.

In addition, the optimal networks were used to predict stiffness for specific targets, and the comparison between the two was illustrated with percent differences (Table 3). The rationale for observing this data was that a network trained well on a dataset generated from the ground truth; computational model should be able to closely predict the target stiffness values when presented with the corresponding kinematics. This rationale was validated, particularly in the case of the feedforward network which predicted all of the target ligament stiffness data within 6% of the expected values. Most of the stiffness values were also predicted well by the RBFN with ten of the fourteen types of stiffness varying no more than 8.7% from the target values. Of the remaining four values, three varied by approximately 19.4% while one fell just under 30% from the expected stiffness. Interestingly, these percent differences did not necessarily equate to large absolute changes in stiffness. For example, the 30% difference of this fourth value (a component of the spring ligament) represented only a 0.6 N/mm difference from the originally assigned value. While this study alone cannot make definitive conclusions regarding the role a particular ligament has on specific foot kinematics, it could indicate upon which ligaments to focus further attention. Higher percent differences may indicate the necessity to generate more input-outputs for network training such that the ANN can better learn the characteristics of the foot/ankle system.

Generally, ANNs require extensive training data [5]; however, the extent may be defined differently depending on the application as no definitive value is specified. Here, during the generation of training data, stiffness variations were adjusted in fixed increments and in combinations (e.g., varying two ligament groupings while one remained constant) to provide a wide stiffness representation to the neural networks. Future investigations could further expand upon the current work by adjusting stiffness in smaller fixed increments and/or additional combinations to increase the number of training data pairs employed by the networks. This could potentially drive down the error observed during training and by extension the amount of variation observed in stiffness predictions.

Finally, an interesting note relevant to network size may be made. As depicted in Table 1, the FFN resulted in fewer neurons during training. This, in part, is due to the fact that only a maximum of ten hidden neurons was tested with the FFN whereas a significantly larger number of neurons were possible in the case of the RBFN. For the FFN, it was thought that a larger network size, thus more network complexity, would be unreasonable given the number of known input-output pairings. Because the number of centers dictated the placement of neurons in the RBFN, a smaller number of centers could result in larger numbers of neurons. Furthermore, as Beale et al. explain, radial basis function networks survey more localized areas of the input space (hence the need for shape parameters which determine the width of the Gaussian transfer functions that are placed at each center) in comparison to feedforward networks, which use sigmoidal transfer functions [13]. Thus, the phenomenon of more hidden neurons in a RBFN than FFN, as was observed here, is likely not uncommon (though the reverse is not impossible [13]).

## 5. Conclusion

While the FFN's performance was better than that of the RBFN, both networks demonstrated acceptable performance when faced with the task of predicting ligament stiffness for a computational foot/ankle model. Future work could involve the development of additional training data pairs in order to further optimize predictions; however, the study shows promise for the application of predicting soft tissue properties using artificial neural networks for the foot/ankle model and advocates use of similar methodology during the examination and creation of other computational models. With the ability to predict improved inputs for computational joint models, biomechanical knowledge of human joint function may advance further.

## Figures and Tables

**Table 1:** Optimal network parameters.

	Parameters		
	*h*	*k*	*σ*
FFN	9	—	—
RBFN	15	11	0.5

Network parameters for optimal feedforward and radial basis function networks. *h* = hidden neurons; *k* = number of centers; *σ* = shape parameter.

**Table 2:** Performance of optimized networks.

	MSE		*R*
	Validation	Test	
FFN	0.032	0.086	0.986
RBFN	0.053	0.054	0.980

Performance for optimized feedforward (FFN) and radial basis function (RBFN) networks as assessed by mean square error (MSE) and correlation (*R*); both compared network-predicted stiffness to target values. MSE was calculated on the validation and test subsets, while *R* was calculated for the entire dataset (*N* = 163). (Note: MSE was calculated using standardized input-output data, which fell within the range of [−1,1].)

**Table 3:** Percent differences, ANN-predictions versus target stiffness.

Ligament	Target stiffness (N/mm)	Percent difference	
		FFN	RBFN
Tibiocalcaneal	75.0	0.89%	6.22%
Tibionavicular	5.0	−5.79%	19.44%
Tibiospring 1	7.6	−5.79%	19.44%
Tibiospring 2	25.0	−5.79%	19.44%
Anterior tibiotalar	90.0	2.98%	2.00%
Posterior tibiotalar	117.0	2.98%	2.00%
Talocalcaneal interosseous	33.8	6.32%	6.58%
Plantar fascia 1	30.0	6.14%	3.61%
Plantar fascia 2	45.0	6.14%	3.61%
Plantar fascia 3	37.5	6.14%	3.61%
Plantar fascia 4	15.0	6.14%	3.61%
Plantar fascia 5	150.0	6.10%	2.72%
Spring 1	16.9	3.22%	8.73%
Spring 2	2.3	3.39%	29.90%

Ligament stiffness values for the patient-specific foot/ankle model alongside percent differences between target values and the ANN-predicted stiffness. (Negative percentages indicate a decrease relative to the target stiffness value.) Within the foot/ankle model, multiple linear elements comprise a single ligament; therefore, the components listed in the table represent each uniquely assigned stiffness value.

## References

[1] Haykin S. (2009). *Neural Networks and Learning Machines*.

[2] Hassoun M. H. (1995). *Fundamentals of Artificial Neural Networks*.

[3] Agatonovic-Kustrin S., Beresford R. (2000). Basic concepts of artificial neural network (ANN) modeling and its application in pharmaceutical research. *Journal of Pharmaceutical and Biomedical Analysis*.

[4] Bas B., Ozgonenel O., Ozden B., Bekcioglu B., Bulut E., Kurt M. (2012). Use of artificial neural network in differentiation of subgroups of temporomandibular internal derangements: a preliminary study. *Journal of Oral and Maxillofacial Surgery*.

[5] Basheer I. A., Hajmeer M. (2000). Artificial neural networks: fundamentals, computing, design, and application. *Journal of Microbiological Methods*.

[6] Massie D. D., Curtiss P. S. Neural network fundamentals for scientists and engineers.

[7] Ahmed F. E. (2005). Artificial neural networks for diagnosis and survival prediction in colon cancer. *Molecular Cancer*.

[8] Lu Y., Pulasani P. R., Derakhshani R., Guess T. M. (2013). Application of neural networks for the prediction of cartilage stress in a musculoskeletal system. *Biomedical Signal Processing and Control*.

[9] Eskinazi I., Fregly B. J. Surrogate knee contact modeling using artificial neural networks.

[10] Kaufman J. J., Chiabrera A., Hatem M. (1990). A neural network approach for bone fracture healing assessment. *IEEE Engineering in Medicine and Biology Magazine*.

[11] Rae S. A., Wang W. J., Partridge D. (1999). Artificial neural networks: a potential role in osteoporosis. *Journal of the Royal Society of Medicine*.

[12] Lippmann R. P. (1987). An introduction to computing with neural nets. *IEEE ASSP Magazine*.

[13] Beale M. H., Hagan M. T., Demuth H. B. (2013). *Neural Network Toolbox™, User's Guide R2013b*.

[14] Wettschereck D., Dietterich T. (1992). *Improving the Performance of Radial Basis Function Networks by Learning Center Locations*.

[15] Spratley E. M., Matheis E. A., Hayes C. W., Adelaar R. S., Wayne J. S. (2013). Validation of a population of patient-specific adult acquired flatfoot deformity models. *Journal of Orthopaedic Research*.

[16] Deland J. T., De Asla R. J., Sung I.-H., Ernberg L. A., Potter H. G. (2005). Posterior tibial tendon insufficiency: which ligaments are involved?. *Foot and Ankle International*.

[17] Saltzman C. L., Nawoczenski D. A., Talbot K. D. (1995). Measurement of the medial longitudinal arch. *Archives of Physical Medicine and Rehabilitation*.

[18] Coughlin M. J., Kaz A. (2009). Correlation of harris mats, physical exam, pictures, and radiographic measurements in adult flatfoot deformity. *Foot and Ankle International*.

[19] Kecman V. (2001). *Learning and Soft Computing, Support Vector Machines, Neural Networks, and Fuzzy Logic Models*.

[20] Chande R. D., Ortiz-Robinson N., Hobson Hargraves R., Wayne J. S. (2015). Application of artificial neural networks to improve predictive ability of computational joint models. *Transactions of the 2015 Annual Meeting of the Orthopaedic Research Society; 2015 March 28–31*.

